# A tumor targeted nano micelle carrying astragaloside IV for combination treatment of bladder cancer

**DOI:** 10.1038/s41598-024-66010-3

**Published:** 2024-07-31

**Authors:** Chenfan Kong, Jianrong Sun, Xinzi Hu, Guangzhi Li, Song Wu

**Affiliations:** 1 Institute of Urology, The affiliated Luohu Hospital of Shenzhen University, Shenzhen University, Shenzhen, 518000 China; 2 Science and Education Department, Shenzhen Traditional Chinese Medicine Hospital, Shenzhen, 518033 China; 3 Department of Oncology, Shenzhen Baoan People’s Hospital, Shenzhen, 518101 China; 4 Department of Oncology, Shenzhen Traditional Chinese Medicine Hospital, Shenzhen, 518033 China; 5 Department of Urology, The Affiliated Shenzhen Hospital of Shanghai University of Traditional Chinese Medicine, Shenzhen, 518009 China

**Keywords:** Nanomedicine, Tumor targeted micelles, aPD-L1, Astragaloside IV, Bladder cancer, Combination therapy, Immunomodulator, Cancer, Urology, Materials science

## Abstract

Immune checkpoint inhibitors (ICIs) are effective agents for tumor immunotherapy. However, their clinical effectiveness is unsatisfactory due to off-target effects and a suppressive immune microenvironment. This study developed a nanodrug delivery system for bladder cancer (BCa) using PCL-MPEG and PCL-PEG-CHO to synthesize internal hydrophobic and external hydrophilic micelles (PP) that encapsulated water-insoluble astragaloside IV (PPA). The aldehyde group on the surface of PPA reacted with the amino group of aPD-L1, allowing the decoration of this antibody on the surface of the micelles. The resultingPPA@aPD-L1effectively piggybacked astragaloside IV and aPD-L1 antibody. These findings suggest that PPA@aPD-L1 is relatively stable in circulation and efficiently binds to BCa cells with the aid of aPD-L1. Additionally, this strategy prolongs the drug’s retention time in tumors. Compared to PBS, PP, and PPA with PPA + aPD-L1 groups, PPA@aPD-L1significantly prolonged the survival of mice with BCa and reduced tumor volume. Mechanistic studies showed that PPA inhibited the NF-κB and STAT3 signaling pathways in tumor cells. Additionally, PPA@aPD-L1increased IFN-γ and decreased IL-10 expression in bladder tumors, affecting the number and type of intratumorally infiltrating T cells. Our study presents a simple and effective drug delivery system that combines herbal monomers with ICIs. It has demonstrated a potent ability to suppress tumor growth and holds potential for future applications.

## Introduction

Tumor immunotherapy has a history of more than 100 years. Recently, research on immune checkpoint inhibitors (ICIs) has made significant breakthroughs, reigniting interest in tumor immunotherapy and making it a hot topic in tumor treatment and research^1^. The most widely researched ICIs have brought hope to many patients with tumors; however, their clinical efficacy remains limited. For example, many patients fail to respond effectively to ICIs. The clinical response rate of anti-PD-1/PD-L1 antibodies against various high-prevalence tumors, such as hepatocellular carcinoma and ovarian cancers were still low^2–4^. This may be attributed to the fact that the response to ICIs is regulated by the immune microenvironment within the tumors, in particular, by the multiple immune cells within the tumor microenvironment^5^. In addition, during treatment with ICIs, the tumor and intratumoral immune microenvironments can transform into immunosuppressive microenvironments, leading to inevitable resistance to ICIs^6^. Therefore, it is crucial to remodel the tumor immune microenvironment for ICIs therapy. In contrast, immunomodulators alter the tumor-suppressive immune microenvironment and enhance the response of immune cells to tumor cells^7–9^. This suggests that the efficacy of ICIs can be increased by combining them with immunomodulators.

Astragaloside IV is a widely used Chinese herbal medicine derived from *Astragali Radix*^10^. According to traditional Chinese medicine (TCM) theory, *Astragali Radix* tonifies qi, which is equivalent to enhancing the body’s immunity. Therefore, *Astragali Radix* is often used in TCM formulas for cancer treatment. Astragaloside IV, the main component of *Astragali Radix*, has a broad spectrum of immune regulatory functions. For instance, astragaloside IV promotes the infiltration and activation of CD8^+^ T cells, reduces regulatory T cell (Treg) infiltration in tumors, and inhibits tumor angiogenesis by regulating multiple signaling pathways, such as STAT3 and NF-κB^11,12^. It has been predicted that the immune regulatory function of astragaloside IV synergistically enhances the therapeutic effect of ICIs, especially in immunosuppressive tumors^13^. Thus, we chose astragaloside IV as an immunomodulator to synergize with anti-PD-L1 antibody (aPD-L1) for bladder cancer (BCa) treatment.

Nano delivery systems have evolved rapidly in recent years. Through the modification of nanocarriers, the application scope of many drugs with low oral bioavailability has been expanded^14^. In addition, nanocarriers have shown significant advantages in drug delivery applications over the past decades. Specifically, nanodrugs have exhibited a propensity to accumulate in tumors via the EPR effect^15^. However, increasing numbers of studies have revealed that relying solely on the EPR effect yields inadequate tumor enrichment of nanodrugs^16^. The efficient delivery of drugs to multiple targets for synergistic therapies poses a challenge due to the highly complex TME^17^. On the contrary, the use of nanocarriers that are endowed with properties of cell-targeting and drug-controlled release through specially designed structures can greatly improve the efficiency of drug delivery in vivo. Therefore, optimizing the efficacy of combined therapy by precisely enhancing cell-targeted release has become an important area of research^18^. The primary approach for designing targeted nanomedicines is to modify nanocarriers with various targeting ligands, including antibodies, peptides, or ligands^19,20^. Several studies suggest that aPD-L1 for ICB can facilitate the targeted binding of nanocarriers to PD-L1-expressing cancer cells and immune cells, thereby enhancing drug delivery to tumor^21,22^. Therefore, with the assistance of nanotechnology, we can deliver astragaloside IV and aPD-L1 to the affected area efficiently and precisely, triggering a strong immune response within the tumor without affecting other organs^23,24^.

Due to their adaptability in the design and modification of their structure and compositions, polymer micelles were frequently employed by researchers in the field of tumor therapy^25^. Polymeric micelles can self-assemble into nano-sized, amphiphilic copolymers in aqueous solution and consist of a hydrophobic core as well as a hydrophilic shell^26^. The hydrophobic core can dissolve the water-insoluble drug while improve its solubility and biostability. Meanwhile, the hydrophilic shell provides micelles with compatibility in aqueous media and protects the drug in the core from interacting with blood components^26^. Polycaprolactone-b-polyethylene glycol (PCL-PEG) micelles have attracted considerable interest as a potential drug carrier. Since their initial discovery, this FDA-approved polymers have been subjected to extensive investigation for a range of biomedical applications, with particular focus on cancer therapy^27^. In addition, various chemical groups were used to modify the PCL-PEG backbone, such as methoxy (PCL-MPEG) and aldehyde group (PCL-PEG-CHO)^28^. These polymers are all highly biodegradable. Therefore, there is no need to worry about the risk of accumulation in the body^27^.

**Figure 1:**
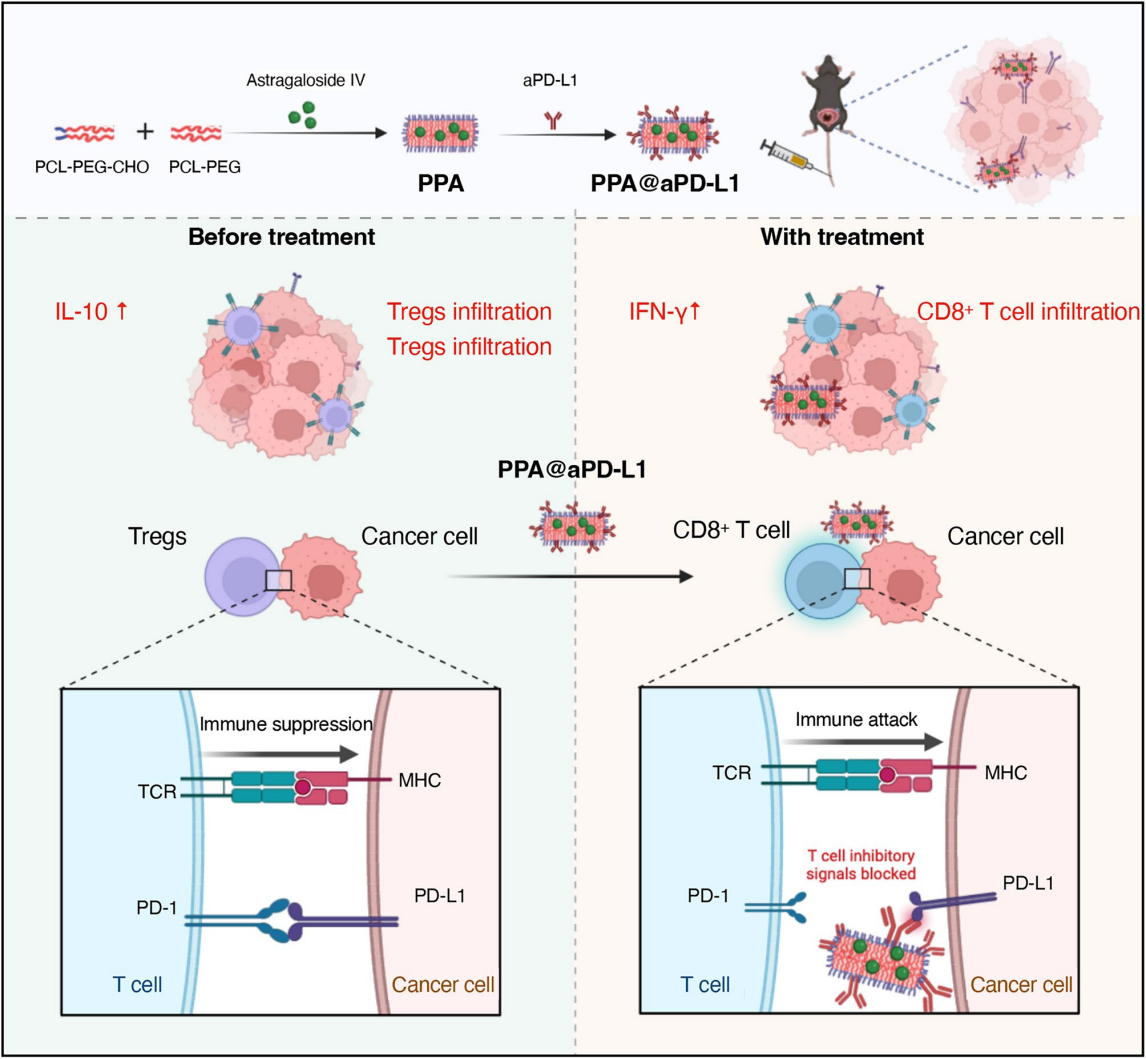
A tumor targeted nano micelle carrying astragaloside IV for combination treatment of bladder cancer.

Based on the above considerations, we utilized PCL-MPEG and PCL-PEG-CHO to synthesize nano micelles (PP) with hydrophilic outer shells and lipophilic inner cores to piggyback on water-insoluble astragaloside IV^29^ (PPA). Moreover, the PD-L1 protein is present in bladder tumors and high expression levels are strongly associated with a poor prognosis^30–33^. Because of the presence of aldehyde groups in the PP shell, it can bind to aPD-L1 (PPA@aPD-L1), thus targeting BCa cells with high levels of PD-L1^30–33^ (Fig. 1). Based on our results, PPA@aPD-L1 had good efficiency in carrying astragaloside IV and aPD-L1 and significantly accumulated in bladder tumors. PPA@aPD-L1 was observed to have satisfactory tumor-suppressive effects and significantly modulated the tumor-immunosuppressive microenvironment in vivo. This study provides a simple and effective strategy for tumor-targeted drug delivery and combination treatment using immunomodulators and ICIs.

## Materials and methods

### Materials and animals

PCL_2K_-MEPG_2k_ and PCL_5k_-PEG_2k_-CHO were purchased from RuiXi Biological Technology (China). Astragaloside IV and acetone were purchased from Aladdin Scientific (China). aPD-L1 (Cat#BE0101) used to synthesize PPA@aPD-L1 (6.6 mg/mL) was purchased from BioXCell (USA). The PE-conjugated anti-mouse PD-L1 antibody (Cat#124,307) for PPA@aPD-L1 visualization was purchased from BioLegend (USA). The antibodies used for flow cytometry, including anti-mouse CD3 antibody (FITC-conjugated, Cat#100,203), anti-mouse CD4 antibody (PE-conjugated, Cat#100,405), anti-mouse CD8a antibody (APC, conjugated, Cat#162,305), anti-mouse CD25 antibody (FITC-conjugated, Cat#101,907), and anti-mouse Foxp3 antibody (Alexa Fluor 488 conjugated, Cat#320,011), were purchased from BioLegend (USA). The antibodies for western blotting and immunofluorescence, including anti-p-STAT3 antibody (Cat#9145), anti-p-p65antibody (Cat#3033), anti-PD-L1 (Cat#60,475), and anti-GAPDH (Cat#2118) antibodies were purchased from CST (USA). Anti-Ki67 (Cat#GB1111141), anti-CD8 (Cat #GB114196), and anti-CD31 (Cat #113,151) antibodies were purchased from ServiceBio (China). Goat anti-rabbit IgG (HRP, Cat#GB23204) and goat anti-rabbit IgG (Cy5, Cat#GB27303) antibodies were purchased from ServiceBio (China). ­ Elisa kits for mouse INF-γ (KE10001) and IL-10 (KE10103) were purchased from Proteintech (China).

DMEM high glucose culture medium and FBS were purchased from Gibco (USA). Trypsin (0.25%) and serum-free freezing medium were purchased from Yeasen Bio (China). DID and Cy5.5 fluorescence dyes were purchased from Beyotime (China).

The mouse BCa cells MB-49 were purchased from the Cell Bank of the Shanghai Institute of Biochemistry and Cell Biology. All cells were cultured in RPMI 1640 or DMEM supplemented with 10% FBS. The cells were maintained at 5% CO_2_ atmosphere and 37 °C.

Male C57BL/6 mice were purchased from Guangdong Yaokang Bio (Guangdong, China). Animal experiments were performed under anesthesia using sodium pentobarbital and were approved by the Animal Care and Use Committee of the Affiliated Luohu Hospital of Shenzhen University, Shenzhen University. All experiments were performed in accordance with relevant guidelines and regulations. The study was reported as described by the ARRIVE guidelines. Besides, the animals were euthanized according to the accepted method of euthanasia as defined by the American Veterinary Medical Association (AVMA) Guidelines on Euthanasia-Approved Euthanasia Method, 2020. The animals were euthanized with sodium pentobarbital (100 mg/kg body weight) intraperitoneally as well as via a secondary method of cervical dislocation.

### Preparation of nanodrugs

Blank PP micelles and astragaloside IV-loaded PPA micelles were synthesized using emulsion solvent evaporation^34^. 8 mg PCL_5K_-PEG_2K_-CHO and 4 mg PCl_2K_-MEPG_2K_ with or without 3 mg astragaloside IV were added to 4 mL of acetone. After 1 h, the agents were completely dissolved, and then 10 mL of deionized water was added to the beaker. After 30 min in an ultrasonic bath, the solution was mixed overnight at room temperature in a fume hood. Acetone was removed through rotary evaporation under a vacuum at 60 °C. PP/PPA was obtained as an aqueous solution. The solution was dialyzed (Mw = 8 kDa) in saline for 24 h and then filtered with a 0.22 μm filter to remove unencapsulated astragaloside IV. The PP/PPA were firstly the samples were placed in a − 80 °C refrigerator for 4 h. Then after the freeze dryer was pre-cooled to below − 50 °C, the samples were placed in a cold trap and dried under vacuum for 12 h. The dried samples were stored in a − 80 °C refrigerator for subsequent High Performance Liquid Chromatography (HPLC), Fourier Transform infrared spectroscopy (FITR) & ultraviolet–visible spectroscopy (UV–VIS) analysis.

The PPA@aPD-L1 was prepared as follows. First, 8 μL aPD-L1 (6.6 mg/mL) was added to 1 mL PPA solution. The solution was mixed at 500 rpm on ice overnight. The uncombined antibody was subsequently removed from the supernatant using ultracentrifugation at 20,000 rpm, 4 °C for 2 h. The white precipitate was PPA@aPD-L1. The precipitate was dried and stored at 4 °C for further use.

### Physicochemical characterization

The content of astragaloside IV in PPA was detected using high performance liquid chromatography (HPLC, LC-2030 3D PLUS, Shimadzu, Japan) at 205 nm (mobile phase: methanol; 0.8 ml/min)^35^. Encapsulation efficiency (EE) % = quality of astragaloside IV in PPA/quality of astragaloside IV added × 100%. Loading capacity (LC) % = quality of astragaloside IV in micelles/quality of micelles × 100%. The surface morphology of the micelles was detected using TEM (acceleration voltage: 200 kV, JEOL 1400, Japan). The particle size, zeta potential and polymer dispersity index (PDI) of the nanodrugs were measured by dynamic light scattering (DLS) using a Zetasizer Nano ZS-90 instrument (Malvern, USA). The stability of PPA@aPD-L1 in serum and stability of micelles at 4 °C were measured by particle size and PDI. The ultraviolet absorption spectrum of astragaloside IV, PP, PPA and PPA@aPD-L1 were detected by UV–VIS Spectro Photometer (UH5300, Hitachi, Japan) with methanol as solvent. Dried PP, PPA@aPD-L1 and free aPD-L1 were used for FTIR test. 1 mg of sample mixed with 100 mg of potassium bromide (KBr), powdered and pressed into thin slices. Then the slices were tested for transmittance on the Fourier transform infrared spectroscope. A pyrene fluorescent probe was added to the PPA solution. The fluorescence intensity at 373–384 nm was detected using a fluorescence spectrophotometer (F-2500, Hitachi, Japan) and was used to calculate the critical micelle concentration (CMC) of PPA. The binding efficiency of the antibodies was measured using the bicinchoninic acid method (BCA)^36^ (Beyotime, China) and polyacrylamide gel electrophoresis (PAGE). PPA was mixed and stirred with an antibody. The mixture was ultracentrifuged at 20,000 rpm for 2 h. The PPA@aPD-L1 sediment was subjected to PAGE with a free antibody as a reference. Unbound antibody was added to the supernatant and used for BCA assays. Binding efficiency % = amount of antibody added − amount of antibody in the supernatant/amount of antibody added × 100%. The PPA@aPD-L1 was dialyzed (MW 200 kDa) in pH 7.4 PBS or pH 6.5 PBS and placed it in a shaker at 37 °C for 48 h. Replaced the outside buffer solution with fresh PBS and analyzed the antibody concentration with BCA method.

### Cytotoxicity and cell uptake

MB-49 cells were seeded into 96-well plates at a density of 9 × 10^3^ per well. After 12 h, the cells were cultured with different concentrations of PP, PPA, and PPA@aPD-L1 for 24 h. Cytotoxicity was measured using the CCK-8 assay. The fluorescent dye Cy5.5 was used as a substitute for astragaloside IV to visualize the micelles and was conjugated with PP. 10^5^ MB-49 cells were seeded in the confocal dish (φ 15 mm, Nest, USA). The adherent cells were then incubated with PP, and the fluorescence intensity was detected at 0, 2, 4, and 6 h using LSCM.

### Western blotting

MB-49 cells were seeded in 6-well plates at a density of 1 × 10^6^ per well. After the cells reached 60% confluence, they were cultured with LPS (500 ng/mL), LPS (500 ng/mL) + PPA (10 μg/mL), or PPA (10 μg/mL) for 24 h. Total protein was extracted using RIPA (ServiceBio, China) with a protease inhibitor cocktail (MCE, USA). After quantification and denaturation, proteins were separated on a 10% gel and transferred onto a PVDF membrane (Millipore, USA). The membranes were blocked with 5% bovine serum albumin (BSA) for 1 h and incubated with primary antibodies at room temperature for 2 h. After washing the membrane with Tris–HCl buffer containing 0.1% Tween-20 (TBST), the membrane was incubated with a secondary antibody at room temperature for 1 h (diluted 1:8000). The membrane was washed again with TBST, and proteins were detected using a chemiluminescence detection system (Bio-Rad, USA).

### Immunofluorescence

MB-49 cells were seeded in 6-well plates at a density of 1 × 10^6^ per well. After the cells reached 60% confluence, they were treated with LPS (500 ng/mL), LPS (500 ng/mL) + PPA (10 μg/mL), or PPA (10 μg/mL) for 24 h. Cells were fixed with 4% paraformaldehyde for 10 min and treated with 0.5% Triton X-100 to penetrate the cell membrane. After washing with PBS, the cells were blocked with 5% BSA at room temperature for 1 h. The cells were then incubated with primary antibody at room temperature for 2 h. After washing with PBS, the cells were incubated with secondary antibody and DAPI for 30 min.

### Establishment of animal model

① Cell preparing: Firstly, the MB-49 cells were digested with trypsin. Secondly, the cells were washed with PBS for 3 times to remove the residual FBS or culture medium. Then, the cells were resuspended in pre cooled PBS with a density of 2.5*10^7^ for future use. ② Establishing BCa model: Firstly, the abdomens of male C57BL/6 mice were shaved and sterilized using an iodophor. Then, opened the abdominal cavity of mice and clamped the apex vesicae carefully with forceps. The 5*10^5^ MB-49 cells was injected into the corpos vesicae using an insulin syringe (30G U-40, BD, USA). After successful injection, a white mass can be seen on the bladder wall (Fig. S4). The abdominal cavity was then sutured and disinfected. All the operations were performed under anesthesia using sodium pentobarbital. Five days after surgery, follow-up treatment and other experiments were performed.

### In vivo tumor accumulation

PPA or PPA@aPD-L1 was decorated with the fluorescent dye DID. Briefly, 10 µL of DID dye (5 nM) was added to 5 mL of PPA or PPA@aPD-L1. The solution was mixed at 500 rpm on ice for 24 h in dark. The solution was then dialyzed (Mw = 8 kDa) in saline at 4 °C for 24 h to remove free DID. After tumor-bearing, 200 μL DID-PPA or DID-PPA@aPD-L1 were injected intravenously. The fluorescence intensity at different time points was detected using a fluorescence IVIS Lumina system (BLT AniView100, Guangzhou Biolight Biotechnology, Guangzhou, China), and the fluorescence intensity of the lower abdomen was determined.

### Anti-tumor activity in vivo

Mice were divided into five groups: PBS, PP, PPA, PPA + aPD-L1, and PPA@aPD-L1 (n = 5). After cancer cell implantation, the mice were treated on days 5, 8, 11, and 14 via intravenous injection. The dose per PPA@aPD-L1injection was 10 mL/kg body weight, which containing 58.44 μg astragaloside IV and 318 μg aPD-L1, according to the CMC, LC% and aPD-L1 binding efficiency%. The PPA group and PPA + aPD-L1 were also given an equivalent dose of the astragaloside IV and aPD-L1. Mice in the PPA + aPD-L1 group were administered the PD-L1 antibody 8 h after PPA injection. Tumor volume = 0.5 × length × width^2^. The tumor levels of IFN-γ, TNF-α, and IL-10 were measured using ELISA. The protein expression of p-p65 and p-STAT3 were measure by western blot. Protease inhibitors and RIPA were added in to the tumor samples. Then, the samples were homogenized by ultrasound under ice bath and centrifuged at 12,000 rpm for 15 min at 4 °C. The following actions of western blot were performed as section “Western blotting”.

### Flow cytometry analysis

 Tumor-infiltrated immune cells were obtained, and the surface antibodies were stained for 45 min. The intracellular FoxP3 was stained after fixation and perforation. The cells were then analyzed using a FACSCalibur (BD, USA). The results were analyzed using FlowJo 10.8.1 (Treestar, USA).

### Immunohistochemical staining

Animal tissues were fixed in 4% paraformaldehyde. Paraffin-embedded tissue sections were deparaffinized using xylene and sequentially rehydrated in anhydrous ethanol and 90%, 80%, and 70% ethanol. The sections were antigenically repaired using an ethylenediaminetetraacetic acid (EDTA) antigen repair solution (1.0 mM, pH 9.0) using the thermal repair method. After washing with TBST, the sections were immersed in 3% hydrogen peroxide solution for 15 min to remove endogenous peroxidase. The primary antibodies were incubated overnight at 4 °C in a wet box (dilution ratio of antibodies: Ki67-1:300; CD31-1:300; CD8-1:1500; Foxp3-1:300). After washing with TBST, the secondary antibody was incubated for 30 min at 37 °C in a wet box (Cat# PV-8000, ZSGB-BIO, China). The tissue sections were then stained with DAB (Cat# PV-8000, ZSGB-BIO, China) and hematoxylin (Cat# BSBA-4021, ZSGB-BIO, China). Sections were dehydrated in 75%, 90%, and 100% ethanol solutions and made transparent using xylene. Finally, the sections were scanned using a digital pathology scanner (KE-PRO-005-EX; KFBIO, China) to capture photographs of the regions of interest.

### ELISA

Tumor samples were weighed and then frozen at -80 °C. 9 μL of pre cooled PBS was added to each mg of sample and homogenized by ultrasound under ice bath. The homogenate was centrifuged at 12,000 rpm for 15 min at 4 °C. The supernatant was used for ELISA detection.

### Statistical analysis

Statistical analyses were performed using one- or two-way analysis ANOVA with Tukey’s test (GraphPad Prism, USA). Data are presented as the mean ± SD, and *P* < 0.05 was considered statistically significant.

### ARRIVE guideline statement

This study is reported in accordance with ARRIVE guidelines.

## Results

### Nanodrug preparation and characterization

**Figure 2:**
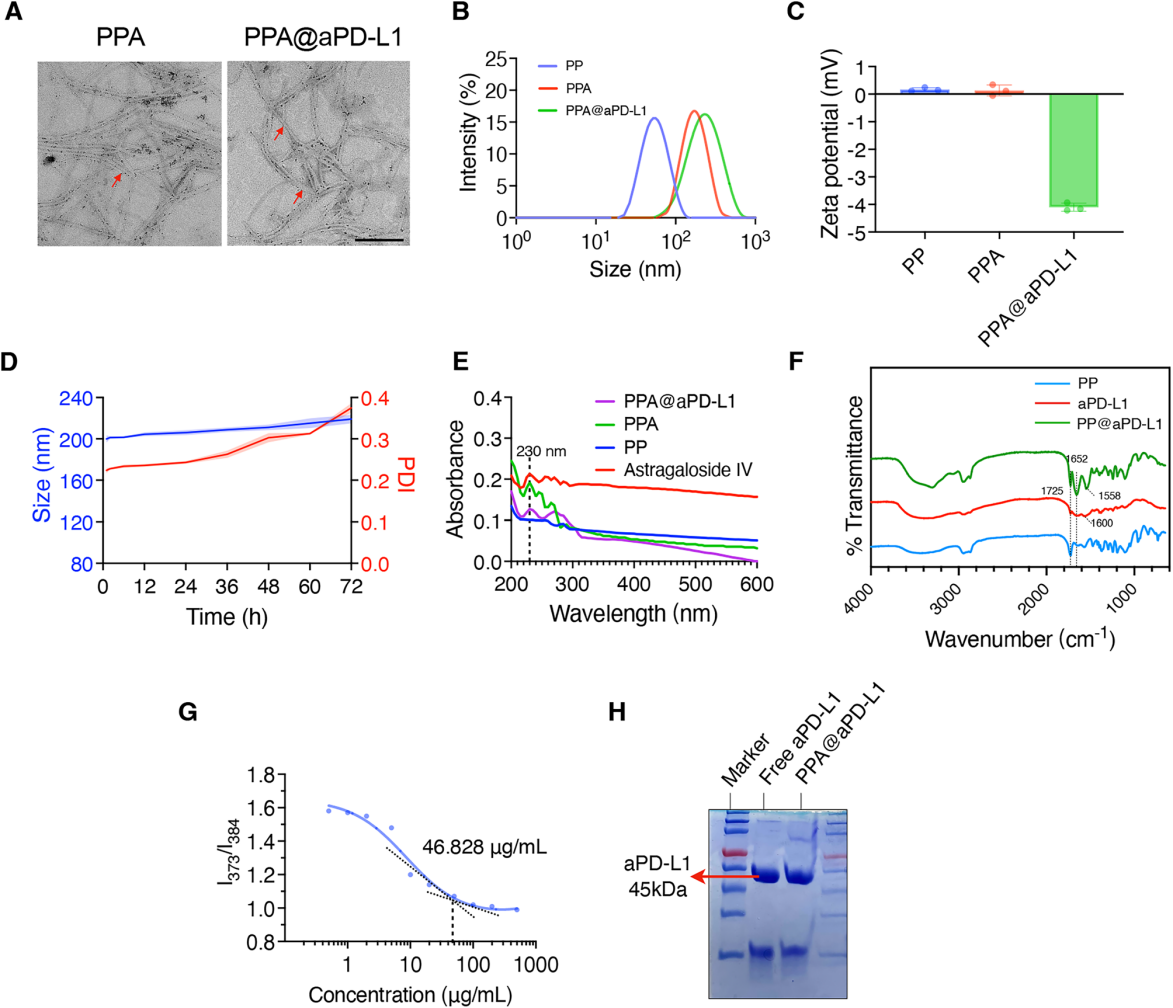
Characterization of nanodrug. (**A**) Transmission electron microscopy (TEM) images of PPA andPPA@aPD-L1(Scale bar = 100 nm). (**B**) The particle sizes of PP, PPA and PPA@aPD-L1determined by dynamic light scattering (DLS). (**C**) The zeta potential of PP, PPA and PPA@aPD-L1determined by DLS (n = 3; means ± SD). (**D**) The particle sizes and polymer dispersity index (PDI) of PPA@aPD-L1 at different times (n = 3; means ± SD) when in the PBS with 10% fetal bovine serum. (**E**) UV–VIS spectrum of FBZ, PP, PPA and PPA@aPD-L1. (**F**) The Fourier transform infrared spectroscopy (FTIR) analysis of PP, aPD-L1 and PP@aPD-L1. (**G**) Critical micelle concentration (CMC) of PPA. (**H**) The SDS–polyacrylamide gel electrophoresis (PAGE) of free aPD-L1 antibody and PPA@aPD-L1 (lane 1: free aPD-L1, lane 2: PPA@aPD-L1).

The rod-shaped nanodrug PPA, which is a piggybacked astragaloside IV, was synthesized using PCL-PEG, PCL-PEG-CHO, and astragaloside IV using a one-pot stirring method. aPD-L1 was then added to the synthetic system and ligated to the aldehyde group on the PPA shell. TEM images (Fig. 2A) show that PPA and PPA@aPD-L1 were rod-shaped. It has been reported that rod-shaped nanomedicines, including rod-shaped micelles synthesized with PEG-PCL polymer, could deliver drugs to tumor more efficiently, in contrast to spherical micelles that were susceptible to be uptaken by monocyte-phagocyte system^37–40^.The particle size of the micelle increased from 62.27 ± 2.86 nm (PP) to 174.03 ± 4.93 nm (PPA) owing to the capsuled astragaloside IV, and the loading of aPD-L1 further increased the particle size to 199.93 ± 2.77 nm (Fig. 2B). As shown in Fig. 2C, PP (0.17 ± 0.05 mV) and PPA (0.13 ± 0.16 mV) were almost electrically neutral, whereas the zeta potential of PPA@aPd-L1 was -4.1 ± 0.12 mV, which was reasonable because of the negatively charged aPD-L1^41–43^. PPA@aPD-L1 was incubated in PBS + 10% fetal bovine serum (FBS), and the particle size and polymer dispersity index (PDI) were measured for 3 days to evaluate its stability in serum. The size of PPA increased from 199.93 ± 2.77 nm to 219.17 ± 3.37 nm after 3 days of incubation in PBS + 10% FBS, demonstrating good serum stability (Fig. 2D). Considering that micelles will be stored at 4 °C for a short time during in vitro and in vivo experiments, we investigated the stability of PP PPA and PPA@aPD-L1 at 4 °C within 48 h (Fig. S1). The results showed that the particle size and PDI of these three formulations did not change much within 48 h, suggesting a good stability at 4 °C. The UV–VIS scanning results showed that the standard astragaloside IV had significant end absorption and characteristic absorption peak at 230 nm. PPA and PPA@aPD-L1 showed an absorption peak at 230 nm, indicating that both PPA and PPA@aPD-L1 contained astragaloside IV. In addition, astragaloside IV showed three obvious absorption peaks at 250-300 nm. The absorption peak of PPA at this area showed blue-shift due to the existence of micelle. The attachment of aPD-L1 to the PPA caused a broad absorption peak at 250–300 nm. (Fig. 2E). In order to demonstrate the interaction between the aldehyde group of the micellar shell and the antibody, we examined the transmittance of PP, aPD-L1 & PP@aPD-L1 by FTIR. According to our results, PP and PP@aPD-L1 showed obvious C=O stretching vibrational absorption peak at 1725 cm^−1^, indicating the presence of aldehyde groups in PP and PP@aPD-L1. aPD-L1 had an obvious N–H bending vibrational absorption peak at 1600 cm^−1^. However, the amine absorption peak of PP@aPD-L1 at this area showed a blue shift (1558 cm^−1^), indicating that the amino group in aPD-L1 was involved in the interaction. In addition, PP@aPD-L1 had a strong absorption peak at 1652 cm^−1^, which was a C=N stretching vibration absorption peak. The above results indicated that part of the aldehyde groups of PP reacted with the amino groups of aPD-L1 thus formed imine bonds (C=N) (Fig. 2F). The content of astragaloside IV in PPA was detected by HPLC. The standard curve of astragaloside IV is shown in Supplementary materials (Fig. S2). The encapsulation efficiency and loading capacity of PPA were 90.92% and 12.48%, respectively. In addition, the critical micelle concentration of PPA was 46.828 μg/mL (Fig. 2G). The aPD-L1 binding rate of PPA@aPD-L1, as detected by BCA, was 60.24% and SDS-polyacrylamide gel electrophoresis (PAGE) provided direct evidence for aPD-L1 binding (Fig. 2H). Furthermore, we imitated the pH inside tumor with pH 6.5 PBS to evaluate the aPD-L1 release rate in a low pH. The result showed that aPD-L1 release form PPA@aPD-L1 continuously, indicating that the aPD-L1 could release and bind to PD-L1 after reaching tumor (Fig. S3).

### In vitro uptake and in vivo targeted delivery of PPA@aPD-L1

**Figure 3:**
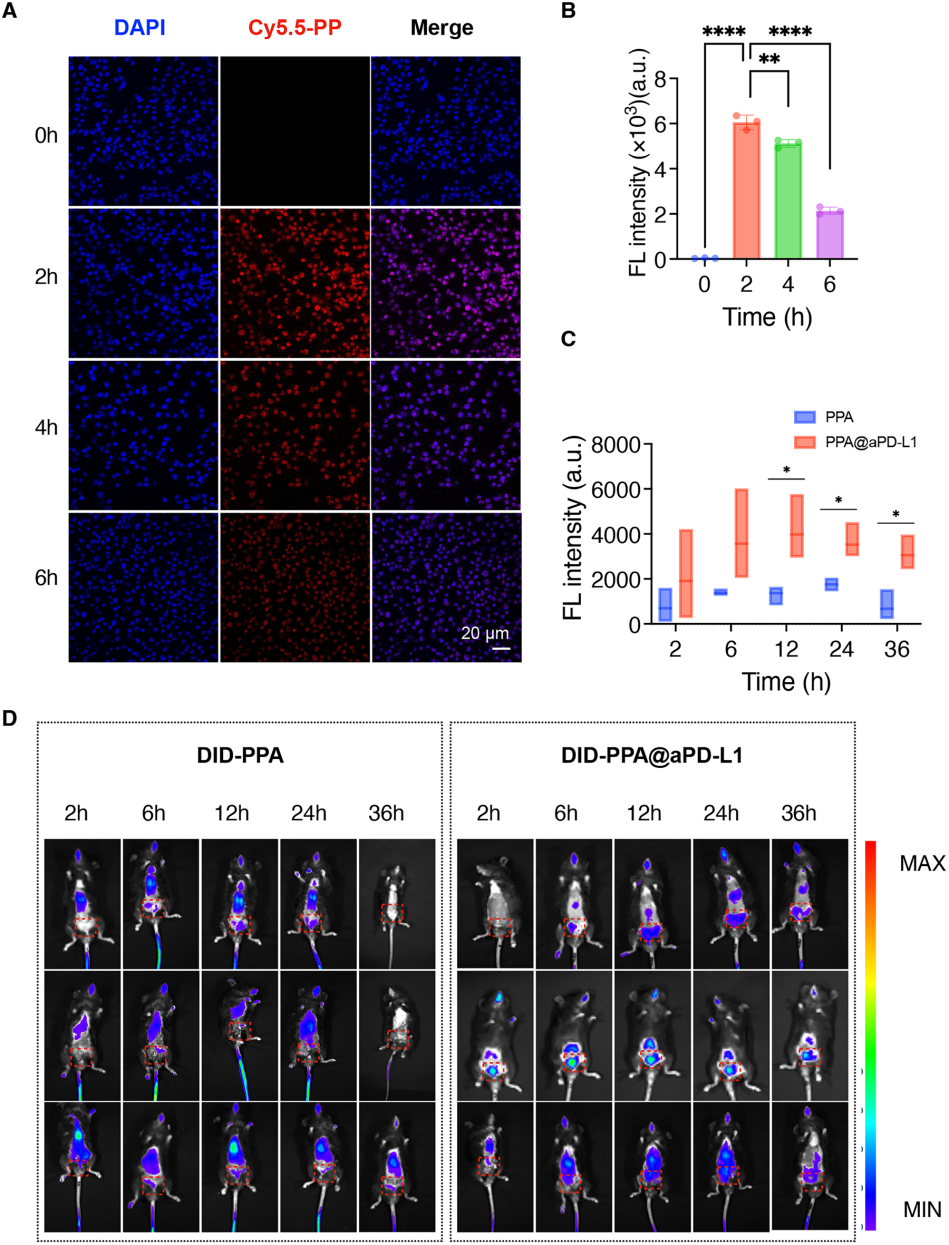
Nanodrugs uptake in vivo and vitro. Laser confocal microscope (CLSM) analysis (**A**) and quantification (B) of MB-49 cells incubated Cy5.5@PPA for different times. Scale bar = 20 μm. (Mean ± SD, n = 3). The quantification of fluorescence intensity (**C**) and images (**D**) of lower abdomen of mice administrated with DID-PPA and DID-PPA@aPD-L1 in vivo (Mean ± SD, n = 3). The location of the tumor was indicated by red box. Statistical significance was determined by one way or ANOVA. *P < 0.05; **P < 0.01; ****P < 0.0001.

A laser confocal microscope (CLSM) was used to assess PPA uptake in vitro. Briefly, the fluorescent dye Cy5.5 was used as a substitute for astragaloside IV. Fluorescence images were captured at 0, 2, 4, and 6 h after the incubation of Cy5.5-PP with BCa cells, which exhibited the strongest fluorescence after 2 h (Fig. 3A,B). We assessed the targeted delivery of PPA@aPD-L1 in vivo. The fluorescent dye DID was bound to PPA and PPA@aPD-L1. After bearing the bladder tumor, the mice were divided into two groups and administered DID-PPA or DID-PPA@aPD-L1. As shown in Fig. 3C,D, fluorescence appeared in the lower abdomen after DID-PPA@aPD-L1 administration, whereas it appeared in the upper abdomen and chest in the DID-PPA group. Furthermore, fluorescence in the lower abdomen of the DID-PPA@aPD-L1 group was stronger than that in the DID-PPA group after 12 h. These results suggested that with the assistance of aPD-L1, PPA@aPD-L1 accumulated much more in bladder tumors than in the lungs or liver. aPD-L1 also prolonged the retention time of the nanodrug in the tumor.

### Inhibition of the STAT3 and NF-κB signaling pathways in vitro

**Figure 4:**
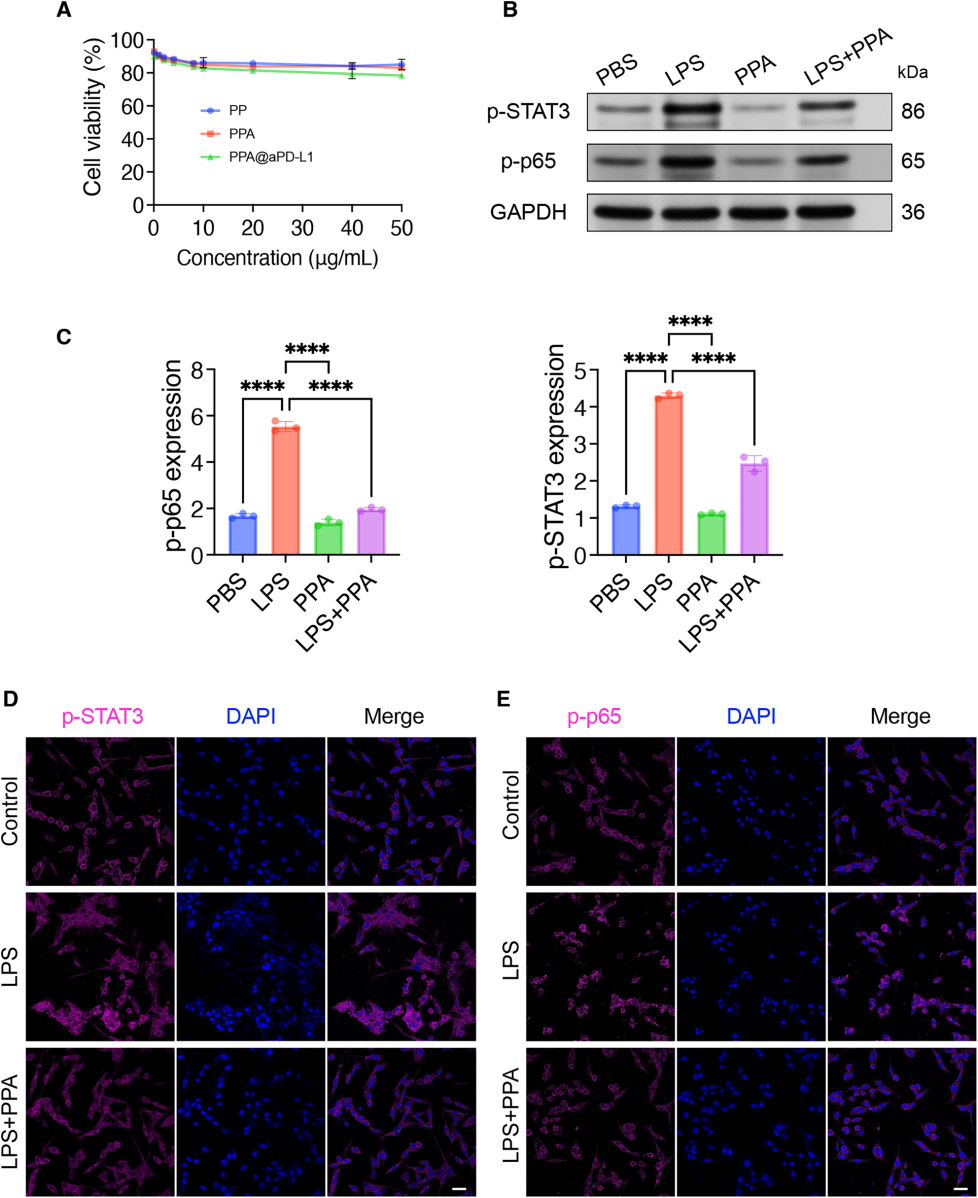
Inhibition of NF-κB and STAT3 pathways of MB-49 cells by PPA. (**A**) Cell viability of MB-49 incubated with different concentration of PP, PPA and PPA@aPD-L1 detected with CCK-8. Protein expression levels (**B**) and quantifications (**C**) of p-STAT3 as well as p-p65 expression in MB-49 cells with GAPDH as reference by western blot (Mean ± SD, n = 3). CLSM images showed the inhibition of nuclear translocation of p-STAT3 (**D**) and p-p65 (**E**) in MB-49 cells incubated with PBS, LPS or LPS + PPA. Both p-STAT3 and p-p65 were labeled with Cy5.5 (purple) and nuclei were stained with DAPI. Scale bar = 20 μm. Statistical significance was determined by one way ANOVA. ****P < 0.0001.

STAT3 and NF-κB are oncogenes associated with drug resistance, metastasis, and angiogenesis. In this study, we found that astragaloside IV was a dual inhibitor of the STAT3 and NF-κB signaling pathways. No obvious cytotoxicity of PP, PPA, or PPA@aPD-L1 was observed indicated a good biosafety in vitro. As a promising immunomodulator, astragaloside IV carried by nano micelles did not exert anti-tumor effects by directly killing tumor cells, but by affecting the protein expression of cancer cells, thus making the tumor microenvironment conducive to immunotherapy. (Fig. 4A). The MB-49 cells were then incubated with LPS, PPA, or LPS + PPA, and the protein expression levels of p-STAT3 and p-NF-κB p65 (p-p65) were measured using western blotting (Fig. 4B). The results showed that PPA significantly decreased the expression of p-STAT3 and p-p65 (Fig. 4C). Finally, we evaluated the nuclear translocation of p-STAT3 and p-p65 in MB-49 cells. As shown in Fig. 4D,E, PPA remarkably reduced the LPS-induced nuclear translocation of p-STAT3 and p-p65 caused by LPS.

### In vivo anticancer effect and immune microenvironment remodeling

**Figure 5:**
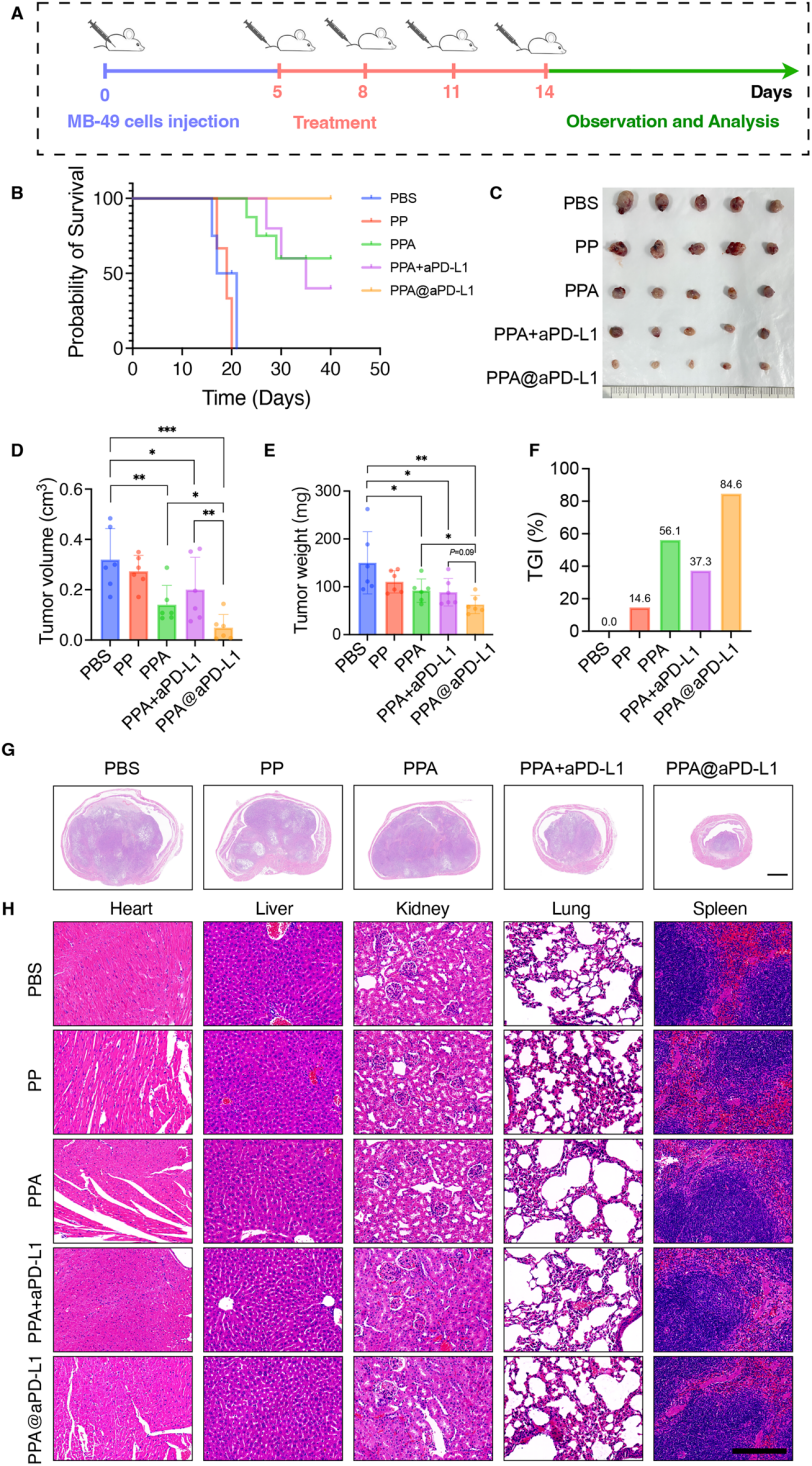
In vivo study of PPA@aPD-L1 against bladder cancer. (**A**) Illustration of in vivo study schedule. PBS, PP, PPA, PPA + aPD-L1 and PPA@aPD-L1 were administered respectively via tail vein 4 times after MB-49 cells implantation. (**B**) Survival of mice receiving different treatments (n = 5). (**C**) The images of tumors of mice receiving different treatments (n = 5). The tumor volume (**D**) and weight (**E**) of mice receiving different treatments (Mean ± SD, n = 5). (**F**) The tumor growth inhibition (TGI) value of mice receiving different treatments (Mean, n = 5). (**G**) The hematoxylineosin (**H**&**E**) staining of tumors of mice receiving different treatments. Scale bar = 1 mm. (**H**) Representative H&E staining images of main organs (heart, liver, kidney, lung and spleen) after treatment. Scale bar = 200 μm. Statistical significance was determined by one way ANOVA. *P < 0.05; **P < 0.01; ***P < 0.001.

Based on tumor-targeting ability and the inhibition of the STAT3 and NF-κB signaling pathways, we then investigated the anti-tumor effect and the immune environment remodeling in the BCa model (Fig. 5A). MB-49 cells were injected in situ to establish the model. Remarkably, treatment with PPA@aPD-L1 significantly prolonged the survival of mice (Fig. 5B), and PPA@aPD-L1 also shrunk the bladder tumors. The mice in the PPA@aPD-L1 group endured minimal bladder tumors in terms of tumor volume and weight. Meanwhile, compared with the PBS group, the tumor growth inhibition value (TGI) of PPA@aPD-L1 group reached 84.6%. (Fig. 5C–F). Hematoxylin and eosin (H&E) staining showed that the mice treated with PPA@aPD-L1 had the smallest tumors, whereas the tumor tissues in the other groups breached the bladder muscularis propria to varying degrees (Fig. 5G). Furthermore, H&E staining of major organs revealed no pathological changes in the sections, suggesting a favorable biosafety profile forPPA@aPD-L1 (Fig. 5H).

**Figure 6:**
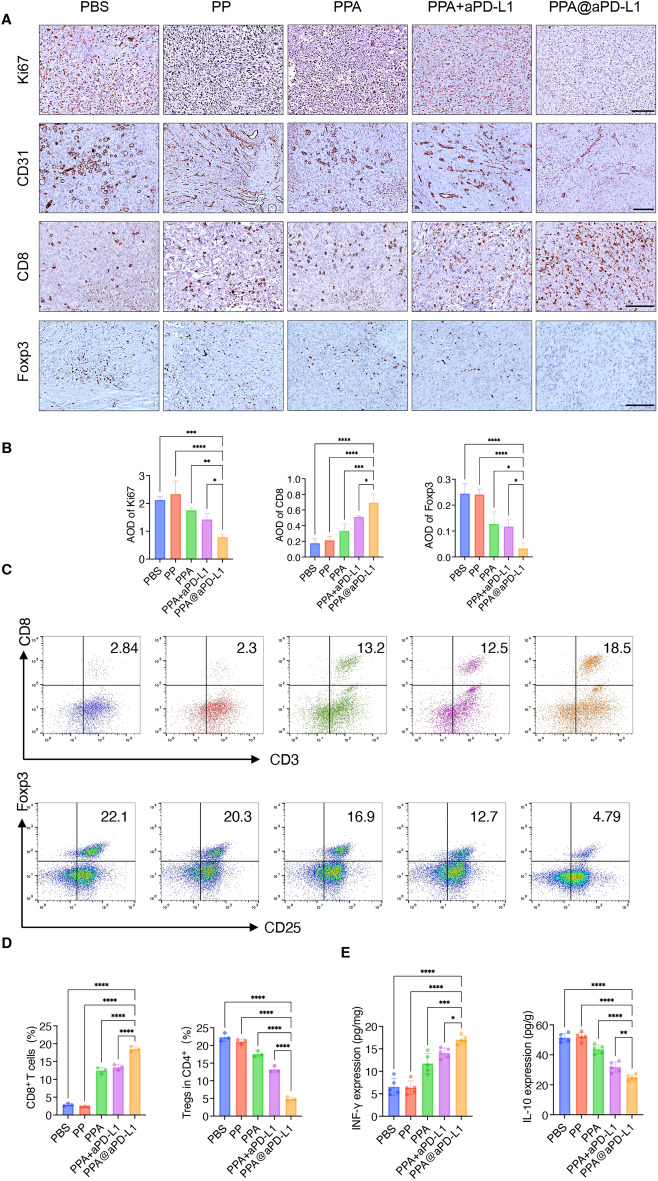
The immunohistochemical staining (**A**) and quantification (**B**) of Ki67, CD31, CD8 and Foxp3 (Mean ± SD, n = 3). Scale bar = 100 μm. Representative plots (**C**) and quantifications (**D**) of CD8^+^ T cell (CD3^+^CD8^+^) and Treg (CD25^+^Foxp3^+^, gated on CD3^+^CD4^+^) in bladder tumor by Flow cytometry (Mean ± SD, n = 3). (**E**) IFN-γ and IL-10 expression of tumors of different treatment groups by ELSIA (Mean ± sd., n = 5). Statistical significance was determined by one way ANOVA. *P < 0.05; **P < 0.01; ***P < 0.001; ****P < 0.0001.

**Figure 7:**
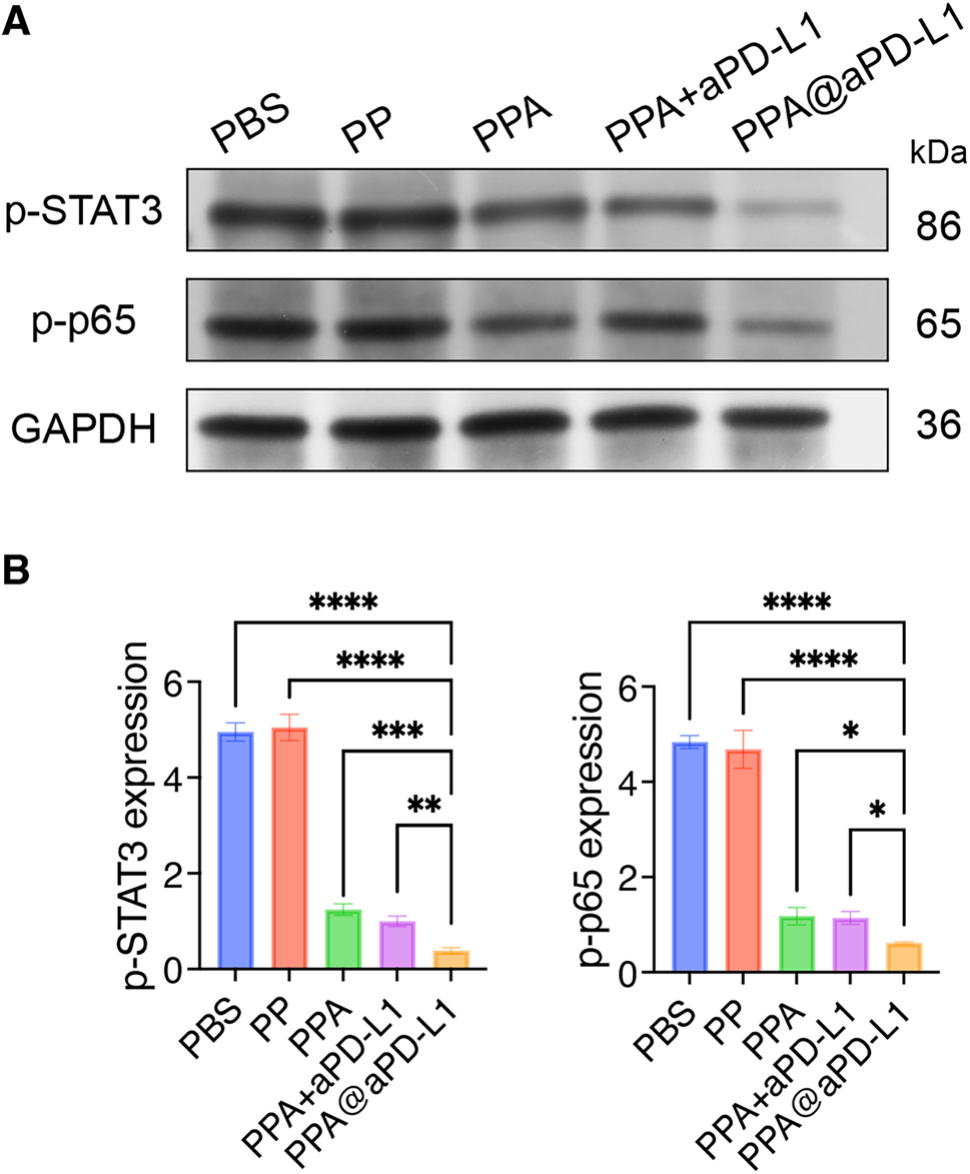
Protein expression levels (**A**) and quantifications (**B**) of p-STAT3 and p-p65 in bladder tumors with GAPDH as reference by western blot (Mean ± SD, n = 3). Statistical significance was determined by one way ANOVA. *P < 0.05; **P < 0.01; ***P < 0.001; ****P < 0.0001.

This study explored the anti-tumor mechanisms of PPA@aPD-L1 and its effects on the immune microenvironment of bladder tumors. The immunohistochemical (IHC) staining results showed that PPA@aPD-L1 remarkably inhibited tumor cell proliferation and reduced angiogenesis (Fig. 6A,B). The infiltration of CD8^+^ T cells and Tregs into the tumor was investigated using IHC and flow cytometry. As shown in Fig. 5A,B, the PPA@aPD-L1 group exhibited more significant CD8^+^ T cell infiltration and less Treg infiltration in tumors than the other groups, which was also supported by the flow cytometry results (Fig. 6C,D). Moreover, IL-10 recruited Tregs^44,45^, and the inhibition of STAT3 and NF-κB reduced the tumor secretion of IL-10^44,46^. IFN-γ recruited and activated CD8^+^ T cells^47^. As shown in Fig. 6E, the expression of IFN-γ increased and that of IL-10 decreased, which revealed the possible mechanisms of PPA@aPD-L1 in remodeling the immune microenvironment. In order to confirm the inhibitory effect of PPA@aPD-L1 on the NF-κB and STAT3 pathways in vivo, we detected the protein expression levels of p-p65 and p-STAT3 in tumor tissues by western blot (Fig. 7A,B). The results indicated that the protein expression levels of p-p65 and p-STAT3 were higher in the tumors of the PBS and PP groups, and this was lower in the PPA, PPA + aPD-L1 and PPA@aPD-L1 groups. This result was consistent with the in vitro experiments (Fig. 4B,C). Furthermore, the protein expression level of p-p65 with p-STAT3 was lowest in the PPA@aPD-L1 group due to active targeting. Taken together with the results of the tumor immune evaluation (Fig. 6), PPA@aPD-L1 remolded the intratumoral immunosuppressive microenvironment by inhibiting the NF-κB and STAT3 pathways in tumor cells.

Notably, based on the anti-tumor and immune environment modification, PPA@aPD-L1 demonstrated a better effect than injecting PPA with aPD-L1 alone (PPA + aPD-L1). These results suggest that our designed nanodrug takes full advantage of tumor-targeted delivery and provides a better strategy for combination treatment.

## Discussion

Currently, multidrug synergistic therapy is the mainstream treatment for tumors. However, combinations of different chemotherapeutic agents are mostly used in clinical practice for BCa^48^. Owing to their low responsiveness and overall survival rates, ICIs are not used in the first-line treatment of BCa but only in advanced disease with metastasis^49^. Considering its mechanism of action, aPD-L1 has significant untapped potential. It is important to acknowledge that tumor immunotherapy based on ICIs requires significant infiltration of anti-tumor T cells^50^. Therefore, the introduction of therapeutic agents that can remodel the tumor immune microenvironment is of great significance in the treatment of tumors using ICIs. To minimize the adverse effects of ICIs, aPD-L1 must be precisely delivered to the tumor.

Astragaloside IV, a widely studied immunomodulator of herbal origin, Astragaloside IV was a very safe herbal compound, was also a potent dual inhibitor of STAT3 and NF-κB according to our results. In addition to NF-κB and STAT3, astragaloside IV could regulate the expression of various signal pathways and growth factors, such as VEGF, Nrf2, HIF-1α, P13k-AKT, etc.^51^, which were closely related to cancer progression, suggesting that astragaloside IV was a promising anticancer drug. But there is still no related to astragaloside IV-related drug in clinical practice at present. In addition, it was likely that the insolubility of astragaloside IV was the reason for the lack of in-depth investigation of its anticancer effects. Despite the publication of over forty papers on this topic, the majority of them have only conducted preliminary investigations in virto^51^. Nevertheless, simply incubating with astragaloside IV made it difficult to analyze whether the drug enters the cells, and did not accurately reflect the complex tumor microenvironment. Moreover, current antitumor experiments in vivo have employed oral administration^51^. However, the bioavailability of astragaloside IV after oral administration was very low^52^. Nevertheless, the bioavailability of astragaloside following oral administration is exceedingly low. In our paper, we demonstrated the precise delivery of astragaloside IV using nano micelles, and visualized the endocytosis by tumor cells. Furthermore, we demonstrated the tumor enrichment of the drug-containing micelles. Moreover, the mechanism by which the low-toxicity astragaloside IV enhanced the efficacy of ICIs by regulating the NF-κB and STAT3 signal pathways had been extensively analyzed. Specifically, IL-10 promotes angiogenesis in the tumor and inhibits cytotoxic T cell activation. In addition, IL-10 induces the overexpression of Foxp3 in CD4^+^ T cells, increasing the number of Tregs in the tumor. Astragaloside IV inhibits the secretion of IL-10 from tumor cells by downregulating the expression of STAT3 and NF-κB in tumor cells. Thus, although astragaloside IV does not exhibit significant tumor cytotoxicity, it can synergistically enhance the therapeutic effect of aPD-L1 by remodeling the immune environment of the tumor.

Nanomaterials are effective tools for tumor drug delivery. To increase the efficiency of aPD-L1 tumor delivery and reduce the adverse effects of the drug, we synthesized the nanomicelle PPA@aPD-L1. Owing to the EPR effect, PPA@aPD-L1 tended to accumulate in the tumors. Subsequently, PPA@aPD-L1, loaded with aPD-L1 on the surface, was delivered to BCa cells because of the high expression of PD-L1 on the cell surface. According to our results, with the aid of aPD-L1, PPA@aPD-L1was retained for a longer period and showed more significant enrichment in tumors than PPA. Additionally, the in vivo antitumor results indicated that PPA@aPD-L1 had better anti-tumor efficacy than injecting PPA with aPD-L1 separately.

In conclusion, the combination of astragaloside IV and aPD-L1 demonstrated excellent anti-tumor efficacy and immune remodeling. This strategy of combining immunomodulators with ICIs expands the scope of ICB applications. Owing to the excellent tumor-targeting ability of the nanodrug, PPA@aPD-L1 can be applied to various types of solid tumors.

### Supplementary Information

Supplementary Information.

## Data Availability

The data used to support the findings of this study are available from the corresponding author upon request.

## Abbreviations

aPD-L1: Anti-PD-L1 antibody
BCa: Bladder cancer
FBS: Fetal bovine serum
ICI: Immune checkpoint inhibitor
PDI: Polymer dispersity index
TCM: Traditional Chinese medicine
Treg: Regulatory T cell
