# Supplementary material for: A tumor targeted nano micelle carrying astragaloside IV for combination treatment of bladder cancer
Source: Sci Rep. 2024 Jul 31;14:17704. doi: 10.1038/s41598-024-66010-3 (PMC11291986; doi:10.1038/s41598-024-66010-3)
Supplement: Supplementary file 1 — Supplementary Information. [file 41598_2024_66010_MOESM1_ESM.docx]

**Supplementary materials**

**A tumor targeted nano micelle carrying Astragaloside IV for combination treatment of bladder cancer**

Chenfan Kong ^1, 2, 3^, Jianrong Sun ^4^, Xinzi Hu ^2, 3^, Guangzhi Li ^2, 3, †^, Song Wu ^1, 3, †^

**Affiliations**

1. Department of Urology, The Affiliated Shenzhen Hospital of Shanghai University of Traditional Chinese Medicine, Shenzhen 518009, China

2. Institute of Urology, The Affiliated Luohu Hospital of Shenzhen University, Shenzhen University, Shenzhen 518000, China

3. Department of Urology, South China Hospital, Health Science Center, Shenzhen University, Shenzhen 518116, China

4. School of clinical medicine, Beijing University of Chinese Medicine, Beijing 100029, China

†Corresponding author: Song Wu (wusong2024@126.com); Guangzhi Li ([sushuzhouyi@sina.com](mailto:sushuzhouyi@sina.com))

Figure S1. The standard curve equation of astragaloside IV.

| A B C |
| --- |

Figure S2. The stability at 4 ℃ of PP (A), PPA (B) and PPA@aPD-L1 (C).

Figure S3. The aPD-L1 release rate at different pH.

| A | B |
| --- | --- |
| 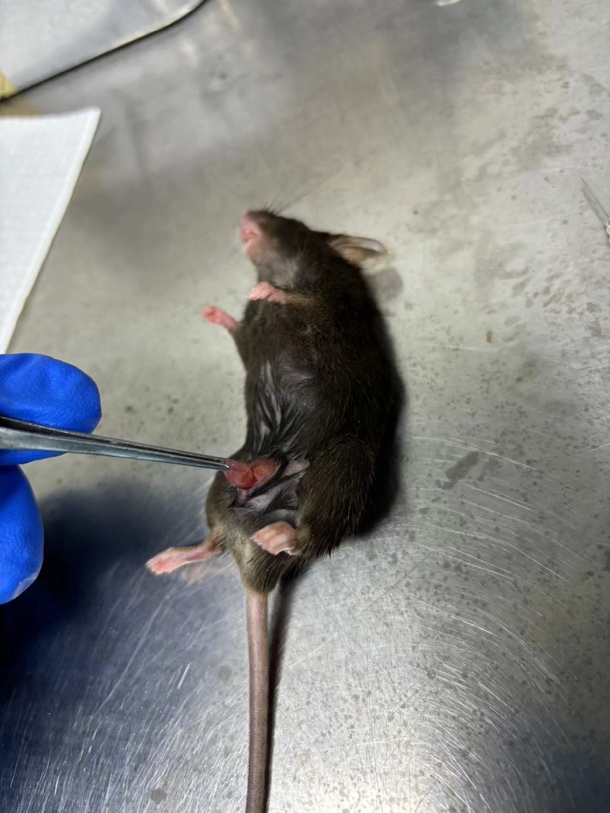 | 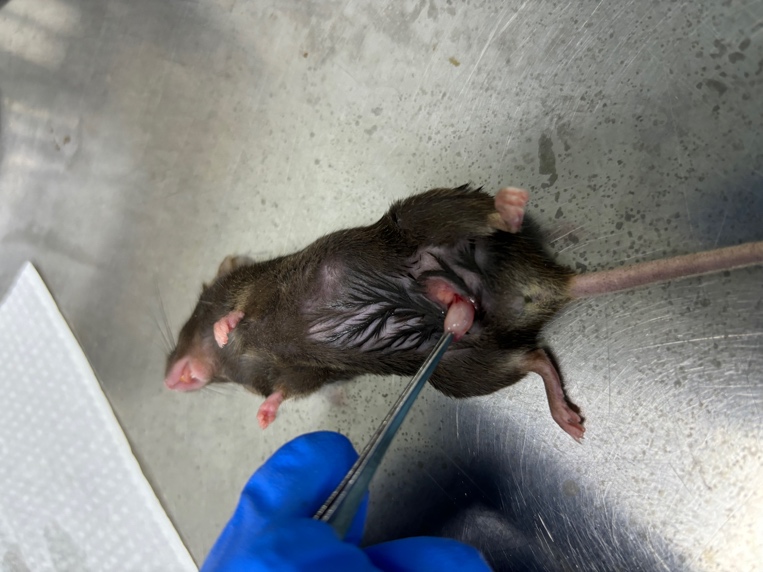 |

Figure S4. Establishment of animal model. (A). Bladder before MB-49 cells injection. (B) Bladder after MB-49 ells injection.
